# Rapid Elution of ^226^Th from a Two-Column ^230^U/^226^Th Generator with Diluted and Buffer Solutions

**DOI:** 10.3390/molecules28083548

**Published:** 2023-04-18

**Authors:** Stanislav V. Ermolaev, Aino K. Skasyrskaya, Aleksandr N. Vasiliev

**Affiliations:** 1 Institute for Nuclear Research of Russian Academy of Sciences, 60-letiya Oktyabrya prospekt 7a, 117312 Moscow, Russia; 2 Department of Chemistry, Lomonosov Moscow State University, GSP-1, Leninskie Gory, 119991 Moscow, Russia

**Keywords:** targeted alpha therapy, alpha-emitter, thorium-226, uranium-230, radionuclide generator, extraction chromatography

## Abstract

A radionuclide generator of the short-lived alpha emitter ^226^Th was proposed. An original scheme consisting of two in-series chromatographic columns was developed for rapidly producing a neutral citric-buffered eluate of high purity ^226^Th. The first column filled with TEVA resin retained the parent ^230^U, while ^226^Th was eluted with 7 M HCl solution to be immediately adsorbed on the second column containing DGA resin or UTEVA resin. Having substituted the strongly acidic medium of second column with neutral salt solution, ^226^Th was desorbed with diluted citric buffer solution. One cycle of generator milking took 5–7 min and produced >90% of ^226^Th in 1.5 mL of eluate (pH 4.5–5.0) appropriate for direct use in radiopharmaceutical synthesis. The ^230^U impurity in ^226^Th eluate was less than 0.01%. The proposed two-column ^230^U/^226^Th generator was tested over 2 months including a second loading of ^230^U additionally accumulated from ^230^Pa.

## 1. Introduction

**Figure 1:**
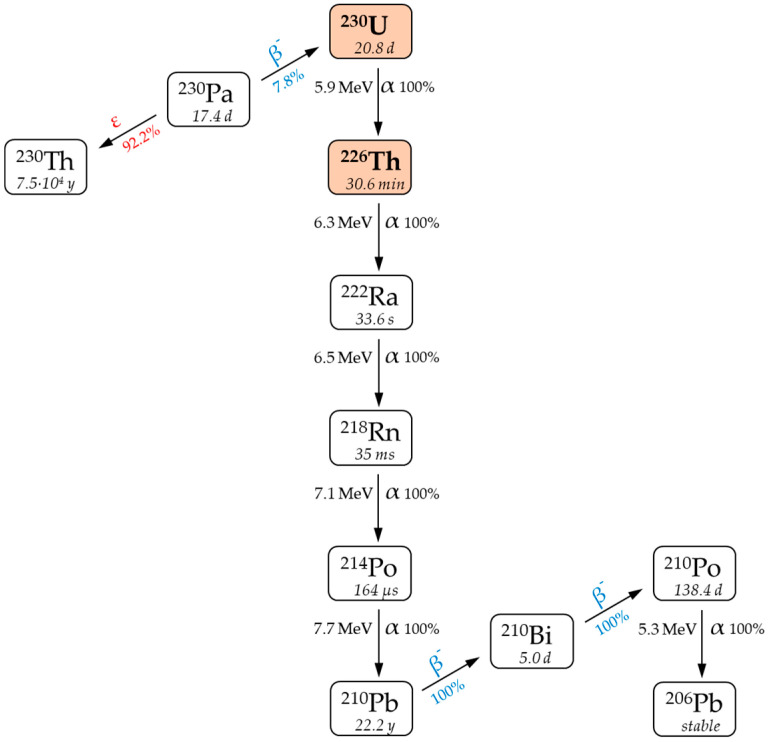
Decay chain of ^230^Pa [2].

A new fast-paced branch of nuclear medicine called targeted alpha therapy (TAT) is effective for the treatment of various oncological diseases due to the property of α-particles to release a large amount of energy in a limited area of living tissue (~10 cell diameters). One of the promising radionuclides for TAT is ^230^U (T_1/2_ = 20.2 d) [1]. The decay of ^230^U generates a chain of short-lived products that emit five α-particles with a total energy of 33.5 MeV (Figure 1), resulting in effective cell damage [2]. The short-lived daughter alpha emitter ^226^Th (T_1/2_ = 30.6 min) is also an attractive radionuclide for using in TAT [3]. In terms of nuclear properties, the ^230^U/^226^Th pair is similar to the well-researched ^225^Ac/^213^Bi generator pair [4].

The expected therapeutic efficacy of ^230^U/^226^Th has not been demonstrated yet; nevertheless, research is underway to find optimal chelating agents that can stably bind in vivo ^230^U [5] and ^226^Th [6]. Daughter nuclide released from a radiopharmaceutical molecule due to recoil effect can potentially migrate from the target cancer cell, which might cause considerable toxic effects to heathy tissues. If the half-life of a formed nucleus is short, its diffusion is time-limited. Therefore, a major advantage of ^226^Th-TAT compared with ^230^U-TAT is that the uncontrolled redistribution of a recoiled daughter nuclide (for ^230^U, this nuclide is ^226^Th itself) is significantly diminished (Figure 1).

The effective and reliable production of ^230^U and ^226^Th is required in order to accelerate biological studies and implement these radionuclides in TAT. ^230^U can be produced directly via nuclear reactions ^231^Pa(p,2n)^230^U [7] and ^231^Pa(d,3n)^230^U [8]. The initial ^231^Pa (T_1/2_ = 3.3 × 10^4^ y) is a decay product of ^235^U, it must be isolated from aged uranium samples. The raw material is hardly accessible, which restricts the method implementation. Another approach uses reactions of thorium nuclei with accelerated protons and deuterons, leading to the formation of the ^230^Pa precursor decaying into ^230^U with a branching ratio of 7.8%: ^232^Th(p,3n)^230^Pa→^230^U and ^232^Th(d,4n)^230^Pa→^230^U.

Scientific organizations worldwide are actively developing the production of ^230^U through irradiation of ^232^Th with protons [9,10,11,12,13]. This method has proven to be the most effective in terms of product yield compared with the reactions with deuterons [14,15]. Moreover, the maximum of the ^232^Th(p,3n)^230^Pa reaction excitation function is around 20 MeV [15,16], which enables up-scaled production of ^230^U on accessible commercial cyclotrons. At higher proton energies (>100 MeV), ^230^U can be obtained as a byproduct in ^225^Ac production [17,18] along with ^223^Ra [19]. 

^230^U from proton-irradiated thorium contains a chemically inseparable impurity of long-lived uranium isotopes. This impurity was evaluated in our previous paper [15] as up to 0.02% ^232^U (T_1/2_ = 68.9 y) and 0.001% ^233^U (T_1/2_ = 1.6 × 10^5^ y). Long-lived admixtures make the medical use of ^230^U as a source in a radionuclide generator of ^226^Th more prospective than direct applications.

^226^Th is considered to be an alternative to another promising generator-produced short-lived radionuclide ^213^Bi (T_1/2_ = 45.6 min) [20]. ^226^Th presumably provides a greater impact on cancer cells compared with ^213^Bi. A rapid cascade of four α-particles initiated by ^226^Th decay deposits totally 27.7 MeV, while ^213^Bi emits only one α-particle with an energy of 8.4 MeV. ^226^Th-radiopharmaceuticals can be effective for therapy of epithelial or easily accessible tumors [21]. Radioimmunoconjugates Nimotuzumab-p-SCN-Bn-DTPA(DOTA) were synthesized in our previous paper and their specificity toward EGFR overexpressing epidermoid carcinoma A431 cells were demonstrated [22]. 

Due to the relatively short half-life of ^226^Th, time economy becomes a major requirement throughout the entire process from obtaining ^226^Th to radiopharmaceutical administration. For this reason, the generator system must ensure the rapid and efficient separation of the accumulated thorium radionuclide. Various methods of liquid–liquid extraction [23,24], extraction chromatography [11,25,26,27], and ion exchange chromatography [28,29,30] have been developed for the separation of thorium and uranium. For example, the methods employing extraction chromatographic sorbents TEVA^TM^ resin and UTEVA/TRU^TM^ resin were recommended for the selective isolation and determination of U, Th, and a number of other radionuclides in water (sample volume up to 1 L) [31,32]. The chromatographic methods are more appropriate for a ^230^U/^226^Th generator since they usually provide ^226^Th in a small volume of eluate containing a reduced amount of long-lived impurities. 

**Figure 2:**
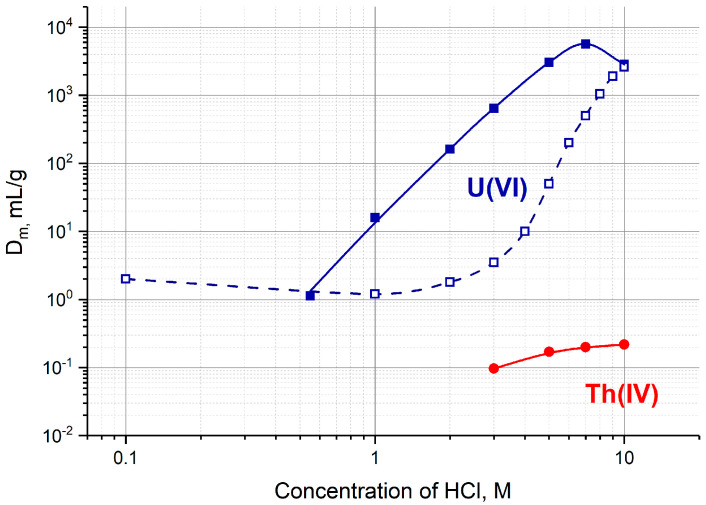
The mass distribution ratios D_m_, mg/mL, of U(VI) and Th(IV) on TEVA resin (solid lines) [31] and Dowex 1 × 10 (dashed line) [33] as a function of HCl concentration.

Effective separation of U(VI) and Th(IV) can be achieved on sorbents displaying anion-exchange properties in strong hydrochloric acid solutions. As it can be seen in Figure 2, U(VI) exhibits high affinity to a strong base anion-exchange resin Dowex 1 (or AG 1) and to an extraction chromatographic resin TEVA at c(HCl) > 6 M, whereas Th(IV) is not retained. Both resins were tested as sorbents for a ^230^U/^226^Th «direct» generator [22]. TEVA resin proved to be more preferable, it provided high ^226^Th yield in less volume of eluate (1–2 mL). Furthermore, the maximal mass distribution ratio D_m_ of U(VI) adsorbed on TEVA resin is located around 7 M HCl, whereas the largest adsorption of U(VI) on AG 1 corresponds to HCl concentration greater than 9 M (Figure 2). The high acidity of the final ^226^Th eluate was found to be the main disadvantage assuming the extra-time needed to convert the eluate into neutral solution prior to labeling. 

Another approach is based on a reverse scheme of a ^230^U/^226^Th generator, i.e., the parent ^230^U is not fixed on the column filled with sorbent while the daughter ^226^Th remains adsorbed. An extraction chromatographic DGA resin containing a diglycolamide derivative was reported to be appropriate for obtaining ^226^Th in citric buffer solution that was amenable to direct labeling with minimal losses of time [13]. However, the ^226^Th eluate recovered from the reported reverse ^230^U/^226^Th generator contained at least 0.2% of ^230^U [13], which was unacceptable for clinical trials to date.

In the presented article, we investigated different schemes of two-column ^230^U/^226^Th generators pursuing two goals: (i) Obtaining ^226^Th of high purity in a solution amenable to further labeling; (ii) reducing the time of ^226^Th production. The first column served for fixing the parent ^230^U and elution of ^226^Th. The second column was intended to adsorb ^226^Th from a strongly acidic solution, and then to desorb it with diluted or neutral solution. This concept was proposed earlier and tested for a ^225^Ac/^213^Bi generator [34,35,36]. In the first column, ^225^Ac formed an extremely strong complex with bis-(2-ethylhexyl)methanediphosphonic acid (H2DEH[MDP]) immobilized on a silica support (Ac resin). Products of the ^225^Ac decay, ^221^Fr, and ^213^Bi were eluted with 1 M HCl and concentrated on the second column filled with the ion exchanger AG-MP 50 from 0.2 M HCl. Then, ^213^Bi was eluted from the second column with 0.1 M HI. The high efficiency of this approach makes it promising for the ^230^U/^226^Th pair, as well.

## 2. Results and Discussion

**Figure 3:**
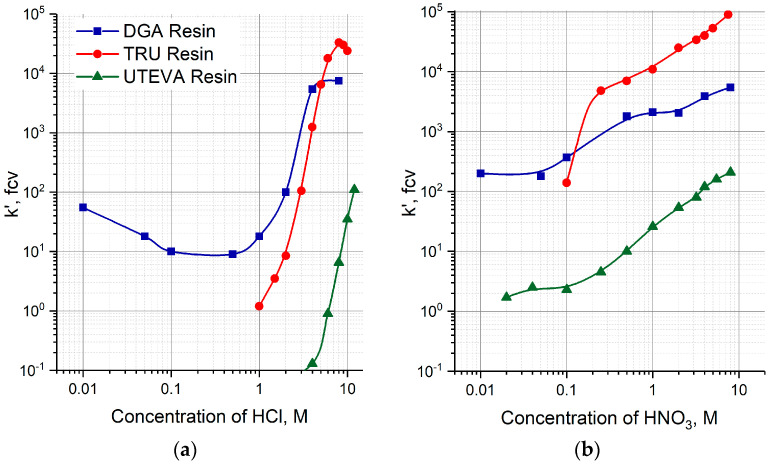
Capacity factors *k*′, fcv, of Th(IV) on DGA, TRU and UTEVA resins as a function of HCl (**a**) and HNO_3_ (**b**) concentrations [37,38,39].

A two-column ^230^U/^226^Th generator was proposed and investigated for fast ^226^Th production in diluted or neutral citric solution. The parent ^230^U was adsorbed onto the first column filled with TEVA resin (Triskem Int.). On reaching the transient equilibrium, the daughter ^226^Th was separated and eluted with strong HCl solution. The role of the second column was to reduce quickly the acidity of ^226^Th solution, i.e., a sorbent for second column was to retain ^226^Th from the strong HCl solution and to desorb it into a diluted solution. Three extraction chromatographic resins eligible for this purpose were considered: TRU resin, UTEVA resin, and DGA resin (all Triskem Int.). According to the reported data [37,38,39] obtained in static conditions and shown in Figure 3a, the resins can be arranged in a row with respect to Th(IV) retention from <5 M HCl solution:

DGA resin > TRU resin > UTEVA resin

In order to evaluate the feasibility of a two-column ^230^U/^226^Th generator, column experiments on ^226^Th sorption from 7 M HCl and its desorption with diluted HCl solutions were carried out.

### 2.1. Elution of ^226^Th from the Second Column with HCl Solutions

**Figure 4:**
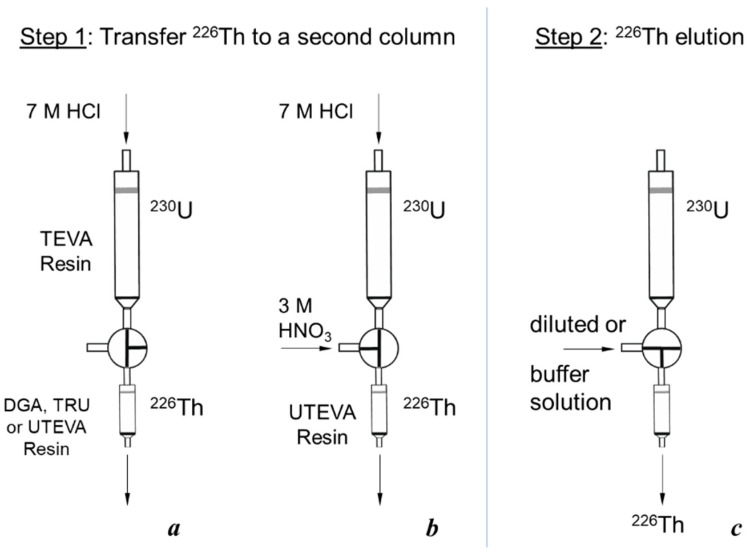
Scheme of ^226^Th production by a two-column ^230^U/^226^Th generator: (**a**,**b**) Two modifications of transferring ^226^Th from the parent ^230^U column to a second one; (**c**) elution of ^226^Th from the second column.

**Figure 5:**
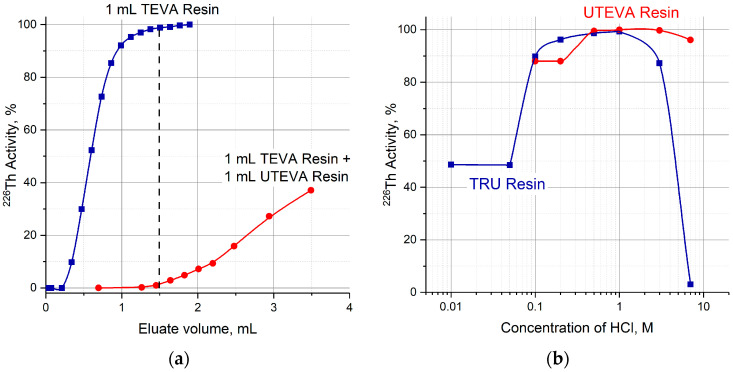
(**a**) Optimization of the UTEVA resin volume in the second column; (**b**) efficiency of ^226^Th desorption from TRU and UTEVA columns as a function of HCl concentration.

First, the transfer of ^226^Th to a second column was investigated (Figure 4a, Step 1). ^226^Th was easily stripped off the parent column with 7 M HCl solution, the concentration corresponding to the maximum of U(VI) sorption on TEVA resin (Figure 2). The integral ^226^Th elution curve (blue line in Figure 5a) indicates that the solution volume of 1.5 mL was sufficient to wash out ≥99% of ^226^Th. DGA resin and TRU resin display high adsorption of ^226^Th from 7 M HCl solution (Figure 3a), the values of *k*′ Th(IV) attain 10^4^. When the second column was filled with 0.1 mL of these resins and connected directly to the exit of the parent column, ^226^Th was completely adsorbed onto the second column. In contrast, the values of *k*′ Th(IV) on UTEVA resin are below 10 under the same conditions. Therefore, the quantity of UTEVA resin in the second column was increased up to 1 mL to ensure a tolerable breakthrough of ^226^Th of less than 3% (red line in Figure 5a).

The transferred ^226^Th was eluted with different HCl solutions as shown in Figure 4c (Step 2). The DGA resin exhibits the greatest retention of ^226^Th among the studied resins from diluted hydrochloric solutions (Figure 3a). Our results of column experiments were in a good agreement with the *k*′ data. The elution of ^226^Th with 0.3 M HCl solution, which is the most favorable for ^226^Th desorption, resulted in 40% of ^226^Th yield in 6 mL of eluate. For other HCl concentrations, the ^226^Th yield was even lower.

**Figure 6:**
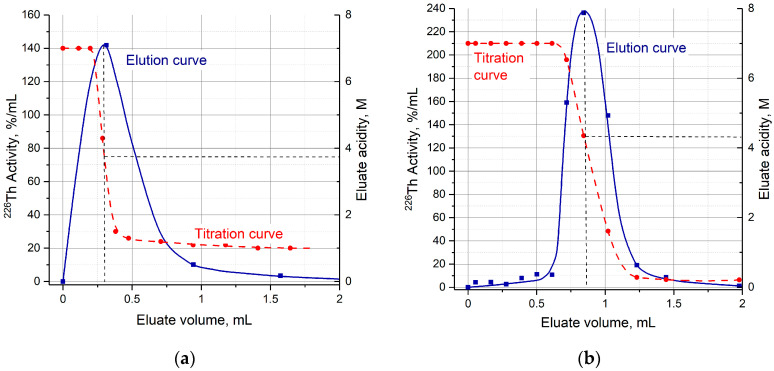
Typical ^226^Th elution and titration curves. (**a**) Elution of ^226^Th from TRU resin with 1 M HCl; (**b**) elution of ^226^Th from UTEVA resin with 0.2 M HCl.

The efficiency of ^226^Th desorption from the second columns filled with TRU and UTEVA resins versus the concentration of hydrochloric solution is presented in Figure 5b. The optimal HCl concentration range of ^226^Th desorption was 0.4–1 M for TRU resin and 0.5–2 M for UTEVA resin. Typical ^226^Th elution curves (Figure 6) display that ^226^Th was completely eluted in ~1 mL of eluate. It is interesting to note that the width of ^226^Th chromatographic peaks from the TRU resin and UTEVA resin columns was almost the same, although the bed volume of UTEVA resin was 10 times larger than the TRU resin.

Despite the fact that the HCl concentration of the solution entering the second column for ^226^Th desorption (Step 2) was relatively low, the eluate acidity from both TRU resin and UTEVA resin columns was 3–4 M [H^+^]. The detailed titration curves of eluate collected by portions (Figure 6) demonstrate that ^226^Th is eluted on the drastic HCl concentration gradient when one solution is replaced by another. The maximum of ^226^Th chromatographic peak corresponds to H^+^ concentration around 4 M. Therefore, the use of dilute HCl solutions allowed us to decrease eluate acidity by only a factor of two.

### 2.2. Elution of ^226^Th from the Second Column with Citric Buffer Solutions

**Figure 7:**
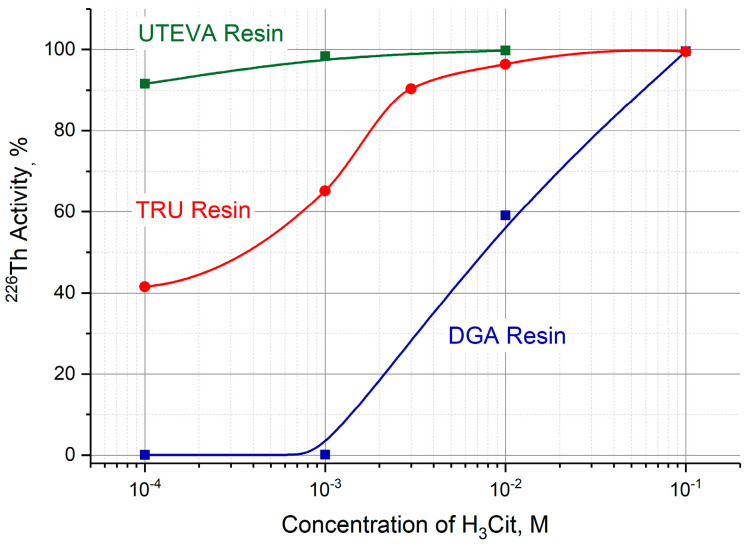
Efficiency of ^226^Th desorption from DGA, TRU, and UTEVA resin columns as a function of citric acid concentration (pH 5.0).

After transferring ^226^Th from the parent column to a second one containing TRU, UTEVA or DGA resin, ^226^Th can be desorbed with a neutral citric buffer solution. The efficiency of ^226^Th desorption was studied as a function of H_3_Cit (pH 5.0) concentration in the range of 10^−4^ M–10^−1^ M. The dependencies plotted on the graph (Figure 7) are arranged in the order reflecting the above-mentioned sequence of resin affinity for thorium (IV). For the studied resins, 0.1 M citric buffer solution fully recovered ^226^Th.

**Figure 8:**
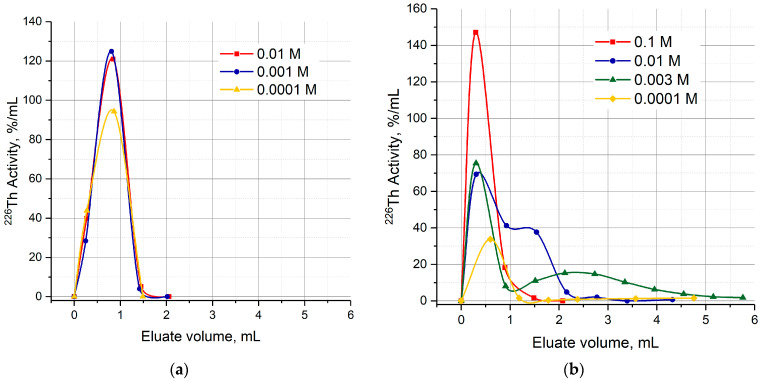
Curves of ^226^Th elution with citric buffer solutions of different concentrations. (**a**) Elution from UTEVA resin; (**b**) elution from TRU resin.

Typical curves of ^226^Th elution from the UTEVA resin and DGA resin columns consisted of the only chromatographic peak within the given range of citric acid concentration (Figure 8a). Similar to the ^226^Th elution with dilute hydrochloric solutions, the peak maximum followed the acidity gradient. Otherwise, two chromatographic peaks were observed when ^226^Th was eluted from the TRU column with citric buffer solutions (Figure 8b). The first peak was in the same way related to the HCl concentration gradient. The position of maximum *V_max_* of the second peak, as well as the capacity factor *k*′ defined as $k^{\prime }=\frac{V_{max}-V_{c}}{V_{c}}$ [37] (*V_c_* is free volume of sorbent in a column), depended on the citric acid concentration.

According to the literature data, various complexes of Th(IV) are coexisting in citric acid media depending on pH values and salinity [40,41]. At pH 5–6, the predominant species are ThCit_3_^5−^ and ThCit_2_(OH)_2_^4−^ [40] with cumulative formation constants (logβ) of 28 and 15, respectively. In more acidic solution (pH < 1), the speciation shifts toward cation forms of Th(IV), e.g., Th^4+^ and ThCit^+^ (logβ = 14). It is evident that all these species may exhibit different affinities to the resins and the detailed analysis is complicated. However, in the case of TRU resin, the dependence of *k*′ Th(IV) on citric acid concentration may be expressed by a simple correlation helpful for practice use:

$$k^{\prime }=\frac{k_{0}^{\prime }}{1+a\cdot \left[H_{3}Cit\right]}$$

where $k_{0}^{\prime }$ and a are empirical constants.

Satisfactory values of ^226^Th yield (>90%) in a small amount of eluate (1–1.5 mL) were obtained for all the studied resins, the eluate characteristics are listed in Table 1.

It was found that eluate acidity remained relatively excessive for immediate synthesis of labeled compounds. In order to maintain ^226^Th on the second column during the substitution of the acidic medium with the neutral one, the influence of nitrate ions was studied.

### 2.3. Stabilization of ^226^Th on the Second Column before Elution

The ability of Th(IV) to form stable anionic complexes with nitrate ions is widely used to separate it from other elements. Comparison of *k*′ values for DGA, TRU, and UTEVA resins reveals higher sorption of Th(IV) from nitric solutions (Figure 3b) than from hydrochloric ones (Figure 3a), especially for the acidity below 1 M. Taking this fact into account, we modified the procedure of ^226^Th production from the two-column ^230^U/^226^Th generator.

The initial part of modification consisted of the pre-treatment of the second column. Before transferring ^226^Th from the parent column (Step 1), 10 mL of HNO_3_ solution of a certain concentration was passed through the second column at a flowrate of 1 mL/min. For the DGA and TRU resins, the HNO_3_ concentration was 0.1 M, which corresponds to moderate values of the capacity factor 150 < *k*′ Th(IV) < 350 (Figure 3b). In the case of UTEVA resin, the values of *k*′ Th(IV) for dilute nitric acid solutions are small; they grow with an increasing acid concentration and reach values around 100 in the region of 3–4 M HNO_3_.

**Figure 9:**
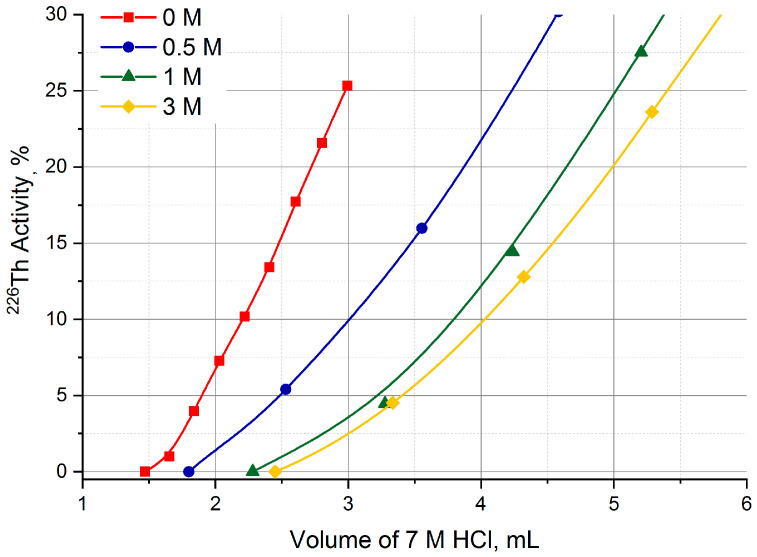
Choice of HNO_3_ concentration for the pre-treatment of UTEVA resin column.

Starting from these data obtained in static conditions, the UTEVA column was pre-treated with a HNO_3_ solution of various concentrations and ^226^Th losses during Step 1 were studied. The results presented in Figure 9 display that the ^226^Th losses when transferring from the TEVA column to UTEVA one with 7 M HCl solution noticeably diminished along with increasing the concentration of HNO_3_ used for UTEVA pre-treatment. For 3 M HNO_3_ solution, the breakthrough of ^226^Th began after passing not less than 2.5 mL of 7 M HCl.

In addition, a variation of Step 1 was tested (Figure 4b) that provided the flow of 7 M HCl solution carrying ^226^Th to be equally mixed with the flow of 3 M HNO_3_ solution at the entrance of the UTEVA column (“two flows” variation). As a result, ^226^Th breakthrough was not observed even after passing 20 mL of 7 M HCl (>20 bed volumes).

**Figure 10:**
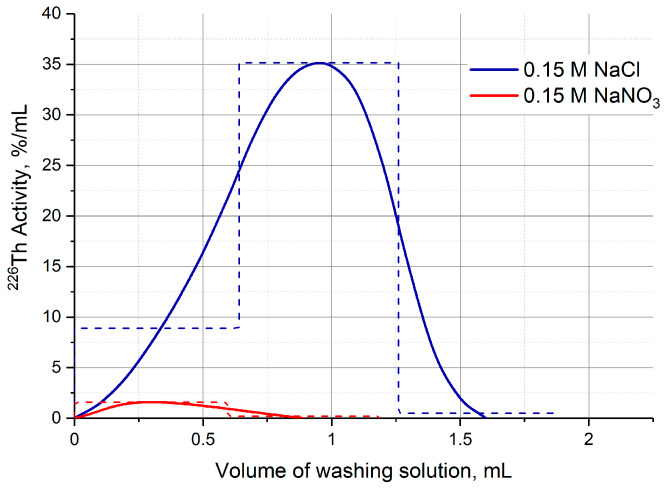
^226^Th losses during the substitution of the 7 M HCl medium on the UTEVA resin column with neutral salt solutions.

The other part of the second column modification was carried out after Step 1 and involved substituting the 7 M HCl medium with dilute or neutral one. Solutions of 0.1 M HNO_3_, 0.15 M NaCl, and 0.15 M NaNO_3_ were studied. Full change in medium in the DGA column took place after passing 1.0–1.2 mL of each solution, and ^226^Th losses did not exceed 3%. In the case of TRU column, ^226^Th was partially washed out on the HCl concentration gradient (similar to Figure 6a) regardless of the substituting solution, its losses ranged from 10% to 40% and were poorly reproducible. The UTEVA resin retained ^226^Th well when the solutions of 0.1 M HNO_3_ and 0.15 M NaNO_3_ were passed through the second column, whereas the 0.15 M NaCl solution washed out up to 28% of ^226^Th (Figure 10).

**Figure 11:**
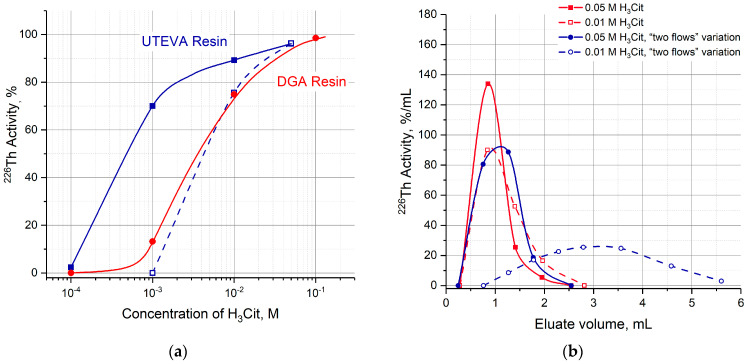
(**a**) Efficiency of ^226^Th desorption from the pre-treated DGA and UTEVA resin columns as a function of citric acid concentration (pH 5.0). DGA resin column pre-treated with 0.1 M HNO_3_ (red solid curve); UTEVA resin: Pre-treated with 3 M HNO_3_ (blue solid curve), pre-treated with 3 M HNO_3_ + “two flows” variation of ^226^Th transfer (blue dashed curve); (**b**) curves of ^226^Th elution depending on the variations of ^226^Th recovery from UTEVA columns.

Having replaced the strongly acidic medium in the second column, ^226^Th was washed out with a citric buffer solution (pH 5.0) of various concentrations (Figure 11a). The results obtained for the UTEVA column:

- without the pre-treatment and medium substitution (green line in Figure 7);
- with the pre-treatment and substitution of 7 M HCl with 0.15 M NaNO_3_ solution (blue solid line in Figure 11a);
- with the pre-treatment, “two flows” variation and the substitution (blue dashed line in Figure 11a)

allow us to suggest that increasing contact time of the UTEVA resin and nitric solution led to an increasing difficult ^226^Th recovery. The relative positions of ^226^Th elution curves (Figure 11b) were also in line with these suggestions.

Moreover, the influence of the contact time was evaluated in parallel experiments that included the 3 M HNO_3_ pre-treatment of the UTEVA column, the HCl substitution with 0.15 M NaNO_3_ solution, and the ^226^Th elution with a 10^−3^ M citric buffer solution. As the pre-treatment duration increased from 10 min to 10 h, the efficiency of ^226^Th production decreased from 70% (see in Figure 11a) to zero. The behavior of the UTEVA resin may be explained by the fact that the *k*′ Th(IV) dependence in nitric solutions is higher than the hydrochloric solutions over the entire concentration range (Figure 3).

Two most effective procedures for obtaining ^226^Th from the two-column ^230^U/^226^Th generator are presented in Table 2.

The overall time of ^226^Th eluate production was within 5–7 min. The time expenditure was slightly shorter when the DGA column was used, since its size was 10 times smaller than the UTEVA column. Meanwhile, the use of UTEVA resin for the second column resulted in high yield of ^226^Th in a less concentrated solution (0.01–0.05 M H_3_Cit, pH 4.5–5.0).

### 2.4. Long-Term Performance of the Two-Column ^230^U/^226^Th Generator

Following the developed procedure, the solution of 7 M HCl was only passed through the parent column containing ^230^U adsorbed on TEVA resin. The distribution of ^230^U along with the length of parent column was monitored throughout the generator lifetime (Figure A2). Usually, two cycles of ^226^Th production per weekday were performed over 2 months. The total volume of 7 M HCl solution that passed through the parent column was about 200 mL including accessory operations and a loading of a second portion of ^230^U. According to the calculations illustrated by Figure A3, one third of the initial amount of ^230^U was additionally accumulated from ^230^Pa in 27–28 days after the first separation, and then loaded onto the parent column.

Due to the two-column scheme of ^226^Th production, the proposed generator provided deep purification ^226^Th from ^230^U. It was found that the ^230^U impurity in the ^226^Th eluates did not exceed 0.01%, which was at least one order of magnitude better in comparison with the reported literature data [13].

In this work, we used low ^230^U activities that do not lead to radiolysis and destruction of the sorbent. Influence of these processes on the generator performance is to be investigated in future studies.

## 3. Materials and Methods

All chemicals were of p.a. (pro analysis) quality or higher, obtained from Merck (Darmstadt, Germany), and used without additional purifications. All experiments were carried out using de-ionized “Milli-Q” water (18 MΩ∙cm^−1^). DGA resin (N,N,N′,N′tetroctyldiglicolamide as an extracting agent), TEVA resin (quaternary ammonium salt Aliquate 336 as an extracting agent), TRU resin (octyl(phenyl)-N,N-di-isobutylcarbomoylmethylphosphine oxide dissolved in tributylphosphate), and UTEVA resin (dipentil pentylphosphonate as an extracting agent) with 50–150 μm particle size were obtained from Triskem, France.

Citric buffer solutions (10^−4^–10^−1^ M, pH 5.0 ± 0.1) were prepared by dissolving the corresponding solid acid sample and adding small portions of 1 M NaOH to obtain a solution with the required pH value. 

Measurements of operational pH values were performed with an Orion 2 Star Benchtop pH meter using an Orion 8103SC combination pH electrode. Commercial pH Titrisol buffer concentrates (Merck p.a.) were used to calibrate the setup at room temperature.

Acid-base titration with indicators methyl orange and phenolphthalein were used to determine the acid content in commercial solutions of concentrated HCl and HNO_3_, as well as in the ^226^Th containing eluate.

The experiments were carried out at a temperature of 21 ± 2 °C.

### 3.1. Gamma-ray Spectroscopy

The measurement of radionuclide activities was performed by γ-ray spectrometry using a high resolution HP Ge detector (ORTEC GEM15P4-70). Samples were counted at different detector-source distances respecting a level of dead-time of less than 10%. The detector efficiency at the used distances were determined with standard calibration sources. Net peak areas in the detected photopeaks were evaluated by means of the GammaVision32 software.

The characteristic γ-ray emission of ^226^Th (111.1 keV, 3.29%) and ^222^Ra (324.3 keV, 2.77%) [2] were used for ^230^U and ^226^Th activity quantification of various generator testing samples respecting the transient equilibrium of daughter radionuclides.

### 3.2. Target Preparation and Irradiation, and ^230^U Isolation

Metallic thorium supplied by Institute for Physics and Power Engineering (IPPE, Russia) was used as target material. Thorium plates of (2.2 × 2.5) cm^2^ approximate dimensions with thickness of 1.5–2.0 mm were fabricated and packed in copper and aluminum foil envelopes, which served for beam monitoring purposes, as well. Each package was encapsulated in a graphite shell sealed with high-temperature silicone adhesive. Several targets were irradiated at the linear proton accelerator of the Institute for Nuclear Research of the Russian Academy of Sciences (INR RAS, Moscow, Russia) [42] with an initial energy of 120–130 MeV. The beam current and total beam charge were 3–5 µA and 12–18 µA·h, respectively.

The dissolution of the irradiated thorium was performed as described previously [18,43] 4 to 5 days after the end of bombardment (EOB). The protactinium fraction including ^230^Pa was recovered from the solution according to the procedure reported in [15] and remained in 7 M HCl/0.1 M HF solution for ^230^U accumulation during 27–28 days. Traces of Nb (mainly ^95^Nb) and Ru (^103,106^Ru) radionuclides were impurities of ^230^Pa/^230^U.

A chromatographic technique close to the one developed by A.W. Knight [44] was implemented for separation of ^230^U from ^230^Pa. The solution with radionuclides was loaded onto a column filled with 2 mL of TEVA resin. Due to the presence of fluoride ions, Pa(V) together with Ru(IV) were eluted, while U(VI) and Nb(V) were retained on the resin. The column was washed with 7 M HCl/0.1 M HF solution to remove Pa(V). The washing was added to the Pa(V) eluate and the combined solution was maintained for the next ^230^U accumulation. Then, having the column washed with 7 M HCl solution, U(VI) and a part of Nb(V) were desorbed with 0.1 M HCl solution. The desorbate was adjusted to 3 M HCl by adding concentrated hydrochloric acid, and following the known procedure [13,45], this solution was passed through a column filled with 1 mL of DGA resin. The uranium fraction was adsorbed, while the ^95^Nb was washed out of the column. Finally, ^230^U was eluted with a small amount of 0.1 M HCl solution.

### 3.3. Generator Schemes for Producing ^226^Th

#### 3.3.1. Preparation of a Parent ^230^U Column

A plastic column of ~5 mm in diameter was filled with 1 mL of TEVA resin equilibrated with 7 M HCl. The resin was fixed inside by two frits at the bottom and at the top of column. The ^230^U solution was evaporated and reconstituted in 7 M HCl. The resulting solution was passed through the column followed by washing with a solution of 7 M HCl. The uranium was adsorbed under these conditions, the loaded activity of ^230^U was 300–350 kBq. Approximately a month after the ^230^U isolation and loading, a second part of ^230^U was accumulated, separated from ^230^Pa as described above and added to the column.

**Figure A1:**
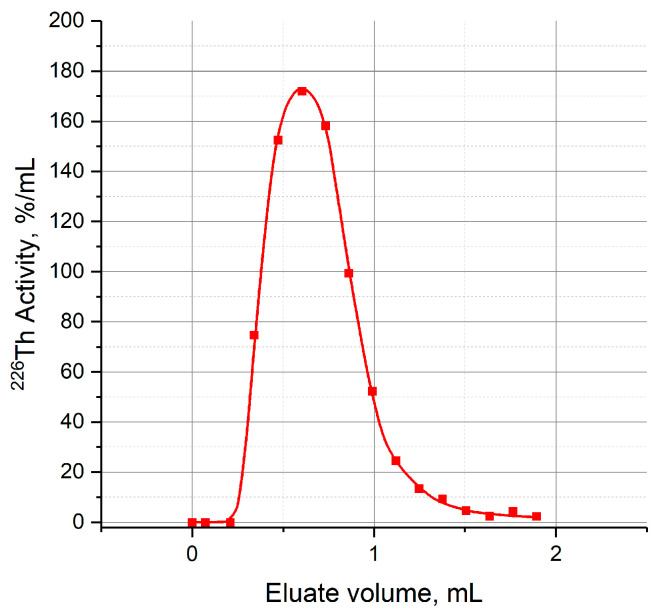
Typical curve of ^226^Th elution from the first column with TEVA resin (1 mL).

**Figure A2:**
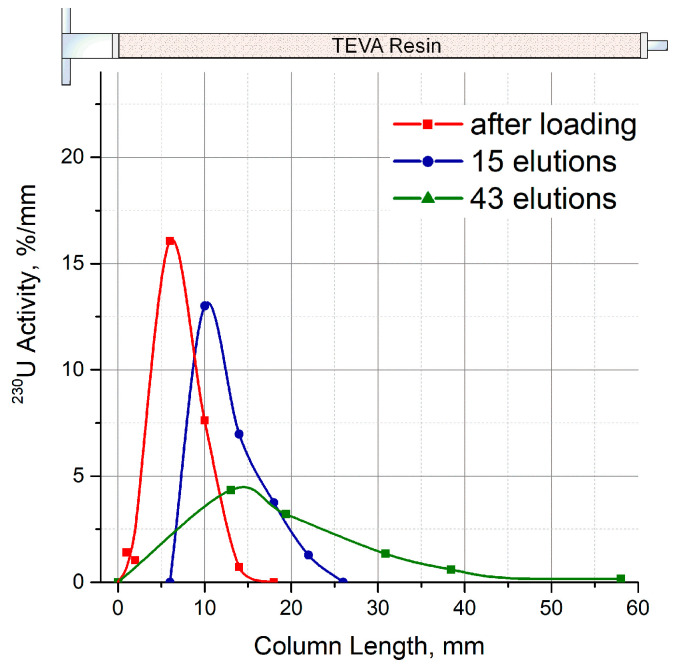
Distribution of ^230^U activity along the length of the first column with TEVA resin depending on the number of elutions.

**Figure A3:**
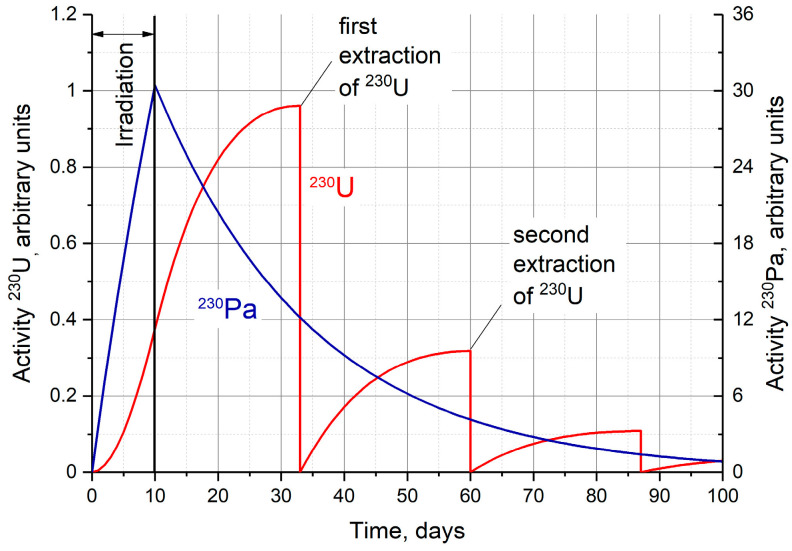
Illustration of ^230^Pa production by a 10-day irradiation of thorium target followed by isolation and periodic extraction of ^230^U (see irradiation details in [15]).

The prepared column served as parent one, and it could work as a one-column generator providing ^226^Th elution in 7 M HCl. A typical differential curve of ^226^Th elution is shown in Figure A1. Furthermore, the parent column was a part of the two-column generator schemes.

#### 3.3.2. Two-Column ^230^U/^226^Th Generator Scheme and ^226^Th Elution Cycle

A general scheme of the ^230^U/^226^Th generator comprised two columns connected in series via a three-valve cock as shown in Figure 4. The parent TEVA column containing ^230^U was the first column and a column filled with TRU, UTEVA or DGA resin served as a second one that could be interchanged when necessary. A milking procedure included two common steps: (1) Transfer ^226^Th with 7 M HCl solution from the parent column to the second one (Figure 4a,b); (2) ^226^Th elution with diluted HCl or citric buffer solution (Figure 4c). Eluates were collected for measurement of ^226^Th and ^230^U activity and eluate acidity. Flow rates of solutions passing through the columns were maintained and controlled with a peristaltic pump. The values of flow rate were 1 and 0.6 mL/min for Steps 1 and 2, respectively.

The TRU or DGA resin was loaded into a plastic column of ~3 mm in diameter, the height of resin bed was 11–12 mm (resin volume ~0.1 mL). Diameter of the column for the UTEVA resin was ~5 mm, the height of the UTEVA bed was 55–58 mm (~1 mL). Each column was equipped with bottom and top frits.

For some experiments with the UTEVA column, we modified Step 1 as it is shown in Figure 4b. A flow of 7 M HCl solution (0.5 mL/min) after passing the parent ^230^U column was mixed with a flow of 3 M HNO_3_ solution (0.5 mL/min) resulting in 1 mL/min flow of 3.5 M HCl/1.5 M HNO_3_ solution at the entrance of the UTEVA resin column.

### 3.4. ^230^U Measurements

The activity of ^230^U in eluates was usually measured overnight for complete ^226^Th decay. ^230^U was assayed by γ-ray spectroscopy via the daughter radionuclides ^226^Th and ^226^Ra.

Distribution of ^230^U along the length of TEVA resin column was monitored by successive scanning of the column through a 4 mm wide slit between lead blocks. The measurements were performed after ^230^U loading onto the TEVA resin column and regularly after milking.

## 4. Conclusions

We have proposed and tested the proof-of-concept of a two-column ^230^U/^226^Th generator for rapidly producing ^226^Th amenable to further labeling. The first ^230^U column with TEVA resin furnished ^226^Th in 7 M HCl solution. The second column retained ^226^Th from the strongly acidic solution, and then released it with a diluted hydrochloric or neutral citric buffer solution. Analysis based on the dependence of the capacity factor *k*′ Th(IV) on the concentration of hydrochloric and nitric acid allowed us to consider DGA, TRU, and UTEVA resins as promising sorbents for the second column.

High yields (>97%) of ^226^Th elution from TRU and UTEVA resins with a small volume (~1 mL) of diluted HCl solutions were obtained. However, the resulting acidity of the eluate was 3–4 M [H^+^] regardless of the solution concentration entering the second column. The titration analysis displayed that ^226^Th was eluted on the HCl concentration gradient when one solution was replaced by another.

Elution of ^226^Th transferred to the second column containing DGA, TRU or UTEVA resin was studied with citric buffer solutions (pH 5.0) in two modes. Direct ^226^Th desorption was also influenced by the acidity gradient. While ^226^Th was stripped off the UTEVA and DGA columns in one chromatographic peak, a typical curve of ^226^Th elution from the TRU column consisted of two chromatographic peaks within the studied range of citric acid concentration. The first peak followed the HCl concentration gradient, and the second one may be attributed to ^226^Th complexation with citrate ions in the course of TRU column elution. Satisfactory yields were achieved by ^226^Th elution from the second column filled with any of the studied resins. For TRU and DGA resins, 1–1.5 mL of 0.1 M H_3_Cit (pH 5.0) solution extracted more than 90% of ^226^Th, while the UTEVA resin column demonstrated similar effectiveness with less concentrated citric buffer solutions (down to 10^−3^ M H_3_Cit). The acidity of citric eluates was about two times lower than the diluted HCl solution’s eluates but still relatively high for immediate labeling.

Neutral citric-buffered ^226^Th eluates were obtained when nitrate ions were introduced. The second column was initially put in contact with a nitric acid solution. Then, after ^226^Th transfer from the parent column, the acidic medium of the second column was substituted with the neutral one maintaining ^226^Th immobile. Solutions of 0.15 M NaCl and of 0.15 M NaNO_3_ were used for the DGA and UTEVA column, respectively. Finally, ^226^Th was extracted with citric buffer solution: 0.1 M H_3_Cit from the DGA column and 0.01–0.05 M H_3_Cit from the UTEVA one. Therefore, one cycle of generator milking took 5–7 min and produced >90% of ^226^Th in 1.5 mL of eluate, pH 4.5–5.0.

The proposed two-column ^230^U/^226^Th generator was tested over 2 months including a second loading of ^230^U additionally accumulated from ^230^Pa. The ^230^U impurity in the ^226^Th eluate was less than 0.01% allowing its use directly in synthesis of radiopharmaceutical compounds.

## Figures and Tables

**Table 1:** Characteristics of ^226^Th eluted from the second column of ^230^U/^226^Th generator with citric buffer solution.

Resin	Concentration of Citric Buffer Solution (Eluent), M	^226^Th Yield, %	Eluate Volume, mL	Eluate Acidity, M
UTEVA	10^−3^–10^−1^	97 ± 2	1.5	1.9–2.1
TRU	10^−1^	94 ± 3	1.2	1.6–1.7
DGA	10^−1^	93 ± 2	1.2	1.6–1.7

**Table 2:** Effective procedures of ^226^Th extraction from the two-column ^230^U/^226^Th generator (>90% of ^226^Th in 1.5 mL of eluate, pH 4.5–5.0).

Step	Solution	
	For DGA Column	For UTEVA Column
Second column pretreatment	0.1 M HNO_3_	3 M HNO_3_
Transfer ^226^Th to a second column	7 M HCl	
Acid substitution in the second column	0.15 M NaCl	0.15 M NaNO_3_
^226^Th elution	0.1 M H_3_Cit, pH 5.0	0.05 M H_3_Cit, pH 5.0

## Data Availability

Not applicable.
